# Prevalence of *Bartonella* spp. by culture, PCR and serology, in veterinary personnel from Spain

**DOI:** 10.1186/s13071-017-2483-z

**Published:** 2017-11-07

**Authors:** José A. Oteo, Ricardo Maggi, Aránzazu Portillo, Julie Bradley, Lara García-Álvarez, Montserrat San-Martín, Xavier Roura, Edward Breitschwerdt

**Affiliations:** 1 Departamento de Enfermedades Infecciosas, Hospital San Pedro-Centro de Investigación Biomédica de La Rioja (CIBIR), C/ Piqueras 98, 26006 Logroño, (La Rioja) Spain; 2 Galaxy Diagnostics, Research Triangle Park, Morrisville, North Carolina USA; 3 Intracellular Pathogens Research Laboratory, Comparative Medicine Institute, College of Veterinary Medicine, North Carolina State University, Raleigh, North Carolina USA; 4 Faculty of Social Sciences, University of Granada, Melilla, Spain; 5 Hospital Clínic Veterinari, Universitat Autònoma de Barcelona, Barcelona, Bellaterra Spain

**Keywords:** *Bartonella* alphaproteobacteria growth medium, *Bartonella henselae*, *Bartonella quintana*, *Bartonella vinsonii berkhoffii*, *Bartonella koehlerae*, Veterinary personnel, Spain

## Abstract

**Background:**

The genus *Bartonella* includes fastidious, facultative intracellular bacteria mainly transmitted by arthropods and distributed among mammalian reservoirs. *Bartonella* spp. implicated as etiological agents of zoonoses are increasing. Apart from the classical *Bartonella henselae*, *B. bacilliformis* or *B. quintana*, other species (*B. elizabethae*, *B. rochalimae*, *B. vinsonii arupensis* and *B*. *v*. *berkhoffii*, *B. tamiae* or *B. koehlerae*, among others) have also been associated with human and/or animal diseases. Laboratory techniques for diagnosis (culture, PCR assays and serology) usually show lack of sensitivity. Since 2005, a method based on a liquid enrichment *Bartonella* alphaproteobacteria growth medium (BAPGM) followed by PCRs for the amplification of *Bartonella* spp. has been developed. We aimed to assess culture, molecular and serological prevalence of *Bartonella* infections in companion animal veterinary personnel from Spain.

**Methods:**

Each of 89 participants completed a questionnaire. Immunofluorescence assays (IFA) using *B. vinsonii berkhoffii* (genotypes I, II and III), *B. henselae*, *B. quintana* and *B. koehlerae* as antigens were performed. A cut-off of 1:64 was selected as a seroreactivity titer. Blood samples were inoculated into BAPGM and subcultured onto blood agar plates. *Bartonella* spp. was detected using conventional and quantitative real-time PCR assays and DNA sequencing.

**Results:**

Among antigens corresponding to six *Bartonella* spp. or genotypes, the lowest seroreactivity was found against *B. quintana* (11.2%) and the highest, against *B. v. berkhoffii* genotype III (56%). A total of 27% of 89 individuals were not seroreactive to any test antigen. *Bartonella* spp. IFA seroreactivity was not associated with any clinical sign or symptom. DNA from *Bartonella* spp., including *B. henselae* (*n* = 2), *B. v. berkhoffii* genotypes I (*n* = 1) and III (*n* = 2), and *B. quintana* (*n* = 2) was detected in 7/89 veterinary personnel. PCR and DNA sequencing findings were not associated with clinical signs or symptoms. No co-infections were observed. One of the two *B. henselae* PCR-positive individuals was IFA seronegative to all tested antigens whereas the other one was not *B. henselae* seroreactive. The remaining PCR-positive individuals were seroreactive to multiple *Bartonella* spp. antigens.

**Conclusions:**

High serological and molecular prevalences of exposure to, or infection with, *Bartonella* spp. were found in companion animal veterinary personnel from Spain. More studies using BAPGM enrichment blood culture and PCR are needed to clarify the finding of *Bartonella* PCR-positive individuals lacking clinical symptoms.

**Electronic supplementary material:**

The online version of this article (10.1186/s13071-017-2483-z) contains supplementary material, which is available to authorized users.

## Background

*Bartonella* spp. are fastidious, facultative intracellular, pleomorphic Gram-negative bacilli included in the class Alphaproteobacteria with at least 35 validated species and three subspecies (http://www.bacterio.net/bartonella.html). A wide range of animals are hosts and reservoirs of *Bartonella* spp., mainly transmitted by feces of fleas and lice, and bites of sand flies and potentially ticks [1].

Although not yet demonstrated, all *Bartonella* species and subspecies should be considered potentially zoonotic. An increasing number of *Bartonella* spp. has been associated with an expanding clinical spectrum in humans and animals [1–3]. Some species, such as *Bartonella bacilliformis* (Oroya fever, Verruga Peruana or Carrión’s disease) are confined to the highlands of Peru, Colombia and Ecuador, as the *Lutzomyia* sand fly vector is geographically limited. *Bartonella quintana*, responsible for trench fever during the first and second World Wars, has reemerged in homeless and alcoholic people with poor hygiene (e.g. refugee camps). *B. quintana* is also a known cause of bacillary angiomatosis and peliosis hepatis in HIV patients, chronic lymphadenopathy in immunocompromised or immunocompetent patients, and blood culture negative (BCN) endocarditis [1].

In 1992, a new bacterium, *Bartonella henselae*, the main agent of cat scratch disease (CSD) was described [4]. Subsequently, *B. henselae* has become the most representative species of the genus and the one most frequently reported as a human or animal pathogen [1]. *Bartonella henselae* is one of the main causes of subacute and chronic lymphadenopathy in children and young people, and also causes other severe infections such as endocarditis, hepatosplenic abscesses, retinopathy, uveitis, peliosis hepatis and bacillary angiomatosis, among others [5]. Other *Bartonella* spp. such as *B. elizabethae*, *B. vinsonii* (subsp. *berkhoffii* and subsp. *arupensis*), *B. koehlerae* and *B. alsatica* have been implicated in endocarditis cases; *B. rochalimae* has been associated with fever and bacteremia; *B. tamiae*, with febrile illness; and *B. grahamii*, with neuroretinitis [1, 6]. For all these reasons, *Bartonella* infections are considered emerging and re-emerging infections in humans.

Although *Bartonella* spp. can grow in axenic media, the sensitivity of culture is usually very low. In recent decades molecular tools have been used diagnostically in clinical practice to test certain samples such as adenopathies or cardiac valves for fastidious organisms. However, these techniques are not routinely available, and serological tests are usually the diagnostic modality offered by microbiological laboratories, although cross reactivity between *Bartonella* spp. may occur [7]. Since 2005, a new method for the diagnosis of *Bartonella* infections has been used for the study of animal and human diagnostic specimens. It is based on an enrichment growth medium for *Bartonella* culture that improves the yield of *Bartonella* spp. detection by subsequent molecular biology techniques (PCR and DNA sequencing), and it is called the *Bartonella* alpha-proteobacteria growth medium (BAPGM) enrichment blood culture/PCR platform [2, 8, 9].

We aimed to investigate culture, molecular and serological prevalence of *Bartonella* spp. infections in a group considered at risk (companion animal veterinary personnel) due to frequent exposure to pets and their associated arthropods.

## Methods

A cross-sectional study was performed to determine the seroprevalence (detection of antibodies to six *Bartonella* spp./subspecies) and bacteremia using enrichment blood culture, PCR and DNA sequencing in companion animal veterinary personnel.

### Subject recruitment

Based on a similar study, the expected prevalence of *Bartonella* DNA in veterinarians was 28% [10]. A minimum sample size of 77 individuals was calculated with a margin of error of 10% and a confidence level of 95%. A sample of 85 veterinarians and five veterinary technicians from different regions of Spain who worked with pets and other wild mammals and who attended the annual national small-animal veterinary congress ‘Vetmadrid 2016’ (2447 attendees) held in Madrid (Spain) in March 2016 were recruited. Before the congress, all attendees were notified about the opportunity to participate in the study. One veterinarian who had recently taken antimicrobials (in the last two months) was subsequently excluded from the study.

### Data and specimen collection

A standardized questionnaire including demographic information, clinical symptoms experienced, occupational and non-occupational domestic and wild animal bites, scratch or exposures, and travel history, was completed. Exposure to, or bites by, different arthropods (i.e. lice, fleas, ticks, mites, bed bugs) was recorded. Persistent/chronic disease was defined in the questionnaire as longer than six months.

Approximately 10–12 ml of blood (5–6 ml in EDTA, 5–6 ml in a serum separator tube) was collected at the time of enrollment. Aseptic technique was used including chlorhexidine decontamination of the skin. An experienced nurse performed venipuncture.

### Specimen processing and diagnostic testing

Refrigerated EDTA-anticoagulated blood and serum samples were transported in less than 48 h by car to the Center of Rickettsiosis and Arthropod-Borne Diseases at CIBIR (Logroño, La Rioja, Spain), where blood was centrifuged and sera stored to -80 °C until being prepared for shipping to Galaxy Diagnostics, Inc., Research Triangle Park, North Carolina, USA.

### *Bartonella* IFA serological testing

To compare results across studies, we used culture, serological and enrichment blood culture/PCR techniques in this study, as were used in a previous study involving veterinary personnel [10]. *Bartonella v. berkhoffii, B. henselae*, and *B. koehlerae* antibodies were determined in the Intracellular Pathogens Research Laboratory (IPRL) at North Caroline State University (North Carolina, USA) using cell culture grown bacteria as antigens and following standard immunofluorescent antibody assay (IFA) techniques. Cell lines were determined *Mycoplasma* free by PCR testing. Canine isolates of *B. v*. *berkhoffii* genotype I (NCSU 93CO-01 Tumbleweed, ATCC type strain #51672), *B. v*. *berkhoffii* genotype II (NCSU 95CO-08, Winnie), and *B. v*. *berkhoffii* genotype III (NCSU 06CO-01 Klara) and feline isolates of *B. henselae* H-1 strain (NCSU 93FO-23 Cisco), *B. henselae* SA2 strain (NCSU 95FO-099, Missy), and *B. koehlerae* (NCSU 09FO-01, Trillium) colonies were passed from agar plate grown cultures into *Bartonella*-permissive cell lines, i.e. the DH82 (a canine monocytoid) cell line for strains *B. henselae* H-1 and SA2, *B. v*. *berkhoffii* I and *B. koehlerae* and Vero cells (a mammalian fibroblast cell line) for *B. v*. *berkhoffii* II and III to obtain antigens for IFA testing. For each antigen, heavily infected cell cultures were spotted onto 30-well Teflon-coated slides (Cel-Line/Thermo Scientific), air-dried, acetone-fixed, and stored frozen. Fluorescein conjugated goat anti-human IgG (Cappel, ICN) was used to detect bacteria within cells using a fluorescent microscope (Carl Zeiss Microscopy, LLC, Thornwood, NY).

Serum samples diluted in phosphate-buffered saline (PBS) solution containing normal goat serum, Tween-20, and powdered nonfat dry milk to block nonspecific antigen binding sites were first screened at dilutions of 1:16 to 1:64. All sera that were reactive at a titer of 64 were further tested with two-fold dilutions out to 1:8192. To avoid confusion with possible nonspecific binding found at low dilutions, a cut-off of 1:64 was selected as a seroreactive titer.

### Growth medium

BAPGM enrichment blood culture/PCR was performed at Galaxy Diagnostics Inc., Research Triangle Park, North Carolina, USA, as previously described [8, 10, 11]. An aliquot of 1 ml of EDTA whole blood was inoculated into 10 ml of BAPGM, after which the cultures were maintained at 35 °C in a 5% CO_2_, water-saturated atmosphere. After 7, 14, and 21-day incubation periods, a 1-ml aliquot of the enrichment culture was inoculated onto blood agar plates and incubated at 35 °C. Plates were checked for colony formation at 7, 14, and 21 days after inoculation.

### Conventional and quantitative real-time PCR analysis

Using standard operating procedures, DNA was extracted from EDTA anticoagulated blood, enrichment liquid blood culture, and from blood agar plate colony isolates if obtained after subculture of the previously enriched blood samples for *Bartonella* spp. PCR [11]. *Bartonella* spp. moreover, strain classification was performed using primers designed to amplify two consensus sequences in the *Bartonella* 16S-23S internal transcribed spacer (ITS) region as described previously with minor modifications [11, 12]. Amplicon size obtained from the 16S-23S ITS region is species dependent, allowing preliminary species identification based on amplicon size. Two sets of oligonucleotides, 325s and 1100as were used as forward and reverse primers for conventional PCR. Meanwhile primers 325s and 538as were used as forward and reverse primers for quantitative PCR along with *Taq*Man probe 438 (Table 1). Additionally, as previously reported [13], PCR screening for *B. koehlerae* was performed using species-specific oligonucleotides Bkoehl-1s and Bkoehl1125as forward and reverse primers, respectively. Amplification of the ITS region at both genus and species (*B. koehlerae*) levels by conventional PCR was performed in a 25 μl final volume reaction containing 12.5 μl of My*Taq* HS Red Mix 2× (Bioline), 0.2 μl of 100 μM of each forward and reverse primer (IDT-DNA Technology), 7.3 μl of molecular-grade water, and 5 μl of DNA from each sample tested. PCR negative controls were prepared using 5 μl of dH_2_O (when testing isolates from plates), 5 μl of DNA from the blood of a healthy dog, or 5 μl of DNA extracted from un-inoculated BAPGM-negative controls (when testing BAPGM enrichment cultures). Positive controls for PCR were prepared by serial dilution (using dog blood DNA) of genomic DNA from *B. henselae* (Houston I strain type) down to 0.001 pg/μl (equivalent to 0.5 bacteria/μL). Conventional PCR was performed in an Eppendorf Mastercycler EP gradient under the following conditions: a single hot-start cycle at 95 °C for 3 min followed by 55 cycles of denaturing at 94 °C for 15 s, annealing at 66 °C for 15 s, and extension at 72 °C for 18 s. Amplification was completed by an additional cycle at 72 °C for 1 min, and products were analyzed by 2% agarose gel electrophoresis with detection using ethidium bromide under ultraviolet light. Amplicon products were sequenced to determine the *Bartonella* sp. and ITS strain type. Quantitative real-time PCR was also performed in a 25 μl final volume reaction containing 12.5 μl of SsoAdvanced Universal Probes supermix (Bio-Rad, Hercules, USA), 0.2 μl of 100 μM of each forward and reverse primer (IDT-DNA Technology, Coralville, USA), 0.2 μl of 100 μM of *Taq*Man probe, and 7.3 μl of molecular-grade water. Positive and negative controls used were as described above. qPCR was performed in a CFX96 Real-time System (Bio-Rad) under the following conditions: a single hot-start cycle at 95 °C for 3 min followed by 44 cycles of denaturing at 94 °C for 10 s, annealing at 66 °C for 10 s, and extension at 72 °C for 10 s. Fluorescence at channel one was detected during the extension cycle. As in conventional PCR, all amplicon products were sequenced to determine the *Bartonella* sp. and ITS strain type. All PCR and un-inoculated BAPGM enrichment controls remained negative throughout the study period.

**Table 1:** Primers and probe used for PCR testing

Oligonucleotide	Type	Sequence (5′–3′)
BsppITS325s	Sense primer	CCTCAGATGATGATCCCAAGCCTTCTGGCG
BsppITS543as	Antisense primer	AATTGGTGGGCCTGGGAGGACTTG
BsppITS1100as	Antisense primer	GAACCGACGACCCCCTGCTTGCAAAGCA
BsppITS438	Taqman probe	FAM-AGGTTTTCC/ZEN/GGTTTATCCCGGAGGGC-IABkFQ
Bkoehl-1s	Sense primer	CTTCTAAAATATCGCTTCTAAAAATTGGCATGC
Bkoehl1125as	Antisense primer	GCCTTTTTTGGTGACAAGCACTTTTCTTAAG

### Sequencing analysis

PCR amplicon sequence analysis was performed using a commercial company (Genewiz, Research Triangle Park, NC). Chromatogram evaluation and sequence alignment were performed using Contig-Express and AlignX software (Vector NTI Suite 10.1, Invitrogen Corp., Carlsbad, CA). Bacteria species and strain were defined by comparing similarities with other sequences deposited in the GenBank database using the Basic Local Alignment Search Tool (Blast v. 2.0), and an in-house curated database.

### Data analysis

Questionnaire data for the study were collected on paper forms, entered into a Microsoft Excel database, and validated comparing the electronic records with the information on the forms. Associations of demographic, risk factor, symptoms and exposure variables were assessed with means and medians for continuous variables, and with counts and percentages in contingency tables for categorical data. Group comparisons were performed. The Fisher's exact test for categorical variables and the Mann-Whitney U-test for numerical variables were used. Data processing was carried out with the R software [14], version 3.3.1 for Windows.

## Results

### Individuals studied

A total of 89 veterinary personnel from different regions of Spain (Madrid: 40; Catalonia: 10; Basque Country: 7; Andalusia: 6; Castilla y León: 6; Valencian Community: 5; Canary Islands: 5; Castilla La Mancha: 4; Galicia: 2; Aragon: 1; Asturias: 1; Navarra 1; and La Rioja: 1) were enrolled in the study and a total of 178 samples (EDTA-blood and sera) and the accompanying questionnaires were used to generate data reported in the study. The main characteristics of the study population (exposures and demographics) are listed in Additional file 1: Table S1. A high percentage of the participants reported having chronic/ persistent non-specific symptoms such as headache, insomnia, fatigue or memory problems.

### *Bartonella* detection

#### Serology

Seroreactivity among the six *Bartonella* spp. or genotypes ranged between 11.2–56.2%, with the lowest percentage reactivity (11.2%) to *B. quintana* (Table 2). Only 24 of 89 (27%) participants were not seroreactive to any test antigen, whereas 13, 13, 14, 14, 9, and 2 participants were seroreactive to 1, 2, 3, 4, 5, or 6 antigens, respectively. Seroreactivity to *B. henselae, B. v. berkhoffii* genotypes I, II and III, and *B. koehlerae* ranged between 28 and 56%.

**Table 2:** Immunofluorescent antibody (IFA) titers to six *Bartonella* spp. or genotypes in the 89 veterinary personnel tested for *Bartonella* exposure. Numerical values represent titers at various dilutions

IFA titer	*Bh*	*Bq*	*Bvb* TI	*Bvb* TII	*Bvb* TIII	*Bk*
< 64	56	79	64	39	54	52
64	16	5	9	30	14	18
128	9	3	11	9	15	9
256	7	2	4	8	5	8
512 or 1024	1	0	1	3	1	2
Reactive^a^ (%)	33 (37.1)	10 (11.2)	25 (28.1)	50 (56.2)	35 (39.3)	37 (41.6%)

*Abbreviations*: *Bh Bartonella henselae*, *Bq Bartonella quintana*, *Bvb TI Bartonella vinsonii berkhoffii* genotype I, *Bvb TII Bartonella vinsonii berkhoffii* genotype II, *Bvb TIII Bartonella vinsonii berkhoffii* genotype III, *Bk Bartonella koehlerae*

^a^Number and % of positives with titers ≥ 64

Among individual study participants, seroreactivity patterns varied among the six *Bartonella* spp. or genotypes used for IFA testing (Table 2). *Bartonella* spp. IFA seroreactivity was not statistically associated with any clinical sign or symptom (all *P*-values > 0.05).

#### BAPGM enrichment blood culture PCR

Six veterinarians and one veterinary technician (7.9%) had a positive BAPGM enrichment blood culture/PCR result (Table 3). There were no significant differences found between these two groups (*P* = 0.343). For PCR-positive participants, *B. quintana* was amplified and sequenced from 14 or 21-day BAPGM enrichment blood cultures (two veterinarians), *B. v*. *berkhoffii* genotype I from 7 and 14-day cultures (one veterinarian), *B. v*. *berkhoffii* genotype III from blood or a 7-day BAPGM enrichment blood culture (one veterinarian and veterinary technician), and *B. henselae* from seven and 14 days BAPGM enrichment blood cultures (two veterinarians). Blood agar plate subculture isolates were not obtained from any participant. No individual was found to be co-infected with more than one *Bartonella* spp. BAPGM enrichment blood culture/PCR positivity was not statistically associated with clinical signs or symptoms (all *P*-values > 0.05; Additional file 1: Table S1). Corticosteroid intake, demographics, and arthropod or animal exposures were not statistically associated with BAPGM enrichment blood culture/PCR positivity (*P*-values > 0.05; Additional file 1: Table S1). Information about exposures, demographics and clinical features of the individuals with BAPGM enrichment blood culture/PCR positivity are summarized in Additional file 2: Table S2.

**Table 3:** PCR/DNA sequencing results from blood and BAPGM enrichment blood cultures for 89 veterinary personnel from Spain

Veterinary personnel (*n* = 89)	Species and strain				
Sample type	*Bh*	*Bvb* TI	*Bvb* TIII	*Bq*	All species
Blood	0	1	1	0	2
7-day culture	0	0	0	0	0
14-day culture	2	0	1	1	3
21-day culture	2	1	0	1	4
Agar plate isolates	0	0	0	0	0
Total (%)	2	1	2	2	7 (7.9%)^a^

*Abbreviations*: *Bh Bartonella henselae*, *Bvb TI Bartonella vinsonii berkhoffii* genotype I, *Bvb Bartonella vinsonii berkhoffii* genotype III, *Bq Bartonella quintana*

^a^Total % bacteremic

### Assays associations (serology and BAPGM-PCR)

One *B. henselae* PCR-positive individual was IFA seronegative to all test antigens, and another one was not seroreactive to *B. henselae*. The remaining five PCR-positive individuals were seroreactive to multiple *Bartonella* spp. antigens (Table 4).

**Table 4:** IFA serological results for bacteremic veterinary personnel that were *Bartonella*-positive by BAPGM enrichment PCR testing

Veterinary personnel (*n* = 89)		IFA titer					
ID	*Bartonella* DNA PCR species	*Bh*	*Bq*	*Bvb* TI	*Bvb* TII	*Bvb* TIII	*Bk*
85459	*Bq*	32	128	32	64	64	64
85504	*Bvb* TIII	256	32	256	256	256	256
85505	*Bvb* TIII	32	< 16	64	64	128	64
85513	*Bh*	< 16	< 16	16	64	16	64
85514	*Bq*	256	256	512	512	512	1024
85515	*Bh*	< 16	< 16	< 16	< 16	< 16	< 16
85533	*Bvb* TI	256	128	128	256	128	256

*Abbreviations*: *ID* identification number, *Bh Bartonella henselae*, *Bq Bartonella quintana*, Bvb *TI Bartonella vinsonii berkhoffii* genotype I, *Bvb TII Bartonella vinsonii berkhoffii* genotype II, *Bvb TIII Bartonella vinsonii berkhoffii* genotype III, *Bk Bartonella koehlerae*

## Discussion

The study presented here documented a high serological (65/89 participants; 73.0%) and molecular (7/89; 7.9%) prevalence of *Bartonella* spp. in Veterinary workers from Spain.

Clinically, the diagnostic interpretation of these serological and molecular test results supporting *Bartonella* exposure or infection, respectively, in patients without typical clinical presentations or environmental epidemiological exposures is challenging. For example, the diagnosis of CSD in a patient with lymphadenopathy after being bitten or scratched by a mammal (cat or dog) is straightforward and microbiological assays are unnecessary in most instances to confirm the clinical diagnosis. In contrast, syndromes or illnesses caused by *Bartonella* spp. such as endocarditis, peliosis, angiomatosis, fever of unknown origin, isolated hepatosplenic abscesses, uveitis, and others are clinically challenging, diagnostically problematic, and require microbiological confirmation of diagnosis. In addition, specifically defining the infecting *Bartonella* sp. as the etiological agent of illness is necessary to determine disease epidemiology and to facilitate disease surveillance among various populations (humans, animals and vectors). However, implicating a *Bartonella* sp. as the causative agent of an atypical clinical presentation can be difficult when using serology, and even if the bacterial DNA is amplified from blood or enrichment blood cultures or if the organism is cultivated from the blood. Recently, *B. henselae* and *B. clarridgeiae* have been isolated from the blood of human donors in Brazil using BAPGM enrichment culture/PCR, indicating that asymptomatic bacteremia can occur in healthy humans [9]. Moreover, long standing *B. quintana* bacteremia has been reported in homeless and in immunosuppressed patients [15]. Thus, it is possible that *Bartonella* spp. can induce long standing intra-erythrocytic and endothelial cell infections in healthy individuals without causing pathology or long standing bacteremia may lead to chronic, slowly progressive vascular injury in an otherwise immunocompetent individual [16]. Data in support of either alternative is minimal. Regardless, *Bartonella* serological or molecular microbiological blood culture results in this study and patients without a medically defined clinical syndrome must be cautiously interpreted.

In most clinical microbiology laboratories the diagnosis of *Bartonella* infections is based on serological assays, with current techniques lacking sensitivity, specificity or both. Moreover, conventional cultures (gold standard) are not routinely used due to the overall poor sensitivity in isolating *Bartonella* spp. In recent years, molecular assays have been used for clinical diagnosis and epidemiological studies in vectors (e.g. fleas) [5, 17–19]. However, the sensitivity of these assays is low when testing clinical specimens, such as blood, joint or cerebrospinal fluid or certain tissues. In this study, a very high prevalence of *Bartonella* spp. antibodies was found using in-house IFA assays and six different species and genotypes and strains. As veterinary personnel have frequent contact with reservoir hosts (cats, dogs, wild and production animals) and with *Bartonella* vectors (fleas, ticks, lice, mites), it is likely that these individuals are repeatedly exposed to these bacteria throughout their careers. Although cross reactivity occurs when IFA is used, exposure to more than one *Bartonella* spp. is also possible in veterinary personnel and others with frequent animal and arthropod exposures. For unknown reasons, the highest seroreactivity in the studied population was observed against *B. v*. *berkhoffii* genotype II, whereas only genotypes I and III were detected by enrichment blood culture/PCR. *Bartonella koehlerae* seroprevalence was also high among veterinary personnel (41.6%), although neither culture nor molecular amplification of this species was obtained in this study, despite testing with *B. koehlerae-*specific PCR primers. The presence of *B. koehlerae* has been reported from the Americas, Europe, Asia and Australia [20–25]. Cats appear to be the primary reservoir hosts for *B. koehlerae*, and this species has been associated with disease in dogs [20–22], with a case of human endocarditis [23] and with a young woman with a longstanding history of hallucinations, sensory neuropathy, and peripheral visual deficits [24]. By serology, *B. koehlerae* was implicated diagnostically in a veterinarian with complex regional pain syndrome type I [25] and as a potential cause or cofactor in the development of arthritis, peripheral neuropathies or tachyarrhythmias [13].

As expected, a high *B. henselae* seroprevalence was found in this veterinary worker population (37.1%). This *Bartonella* has a worldwide distribution, cats are the main reservoirs and vectors, and fleas (*Ctenocephalides felis* and *C. canis*) are considered the primary arthropod vectors for infecting cats and other animals. *B. henselae* is the primary or sole causative agent of CSD and induces other clinical syndromes in humans [1]. Documentation of human *Bartonella* infections is scarce in Spain. Nevertheless, clinical descriptions [5, 17, 26] and seroepidemiological studies about the prevalence of *B. henselae/B. quintana* [27–29] have been reported. In our previous studies carried out in La Rioja (North of Spain), *B. henselae* seroprevalences in cat owners, HIV-infected patients and blood donors were 28.9%, 17.3% and 5.9%, respectively, using a commercial kit [30, 31]. These lower percentages most likely reflect differences in the antigens used for testing, and exposure risks among the study populations, with veterinary personnel, most likely having the highest exposure risk. As previously reported, serological tests may not reliably distinguish between antibody responses to *Bartonella* spp. moreover, other infectious agents [32]. Contact with cats and their fleas are the main epidemiological risk factors that contribute to acquiring *B. henselae* infection, and this species has also been involved in disease in dogs from Spain [33]. In a molecular study carried out in fleas from La Rioja, 3.4% and 6.8% *C. felis* were infected with *B. henselae* and *B. clarridgeiae*, respectively [18]. Importantly, cats and dogs are frequently infested with numerous fleas, rather than a single or few fleas. Both *B. henselae* and *B. clarridgeiae* have been isolated from healthy human blood donors in Brazil [9, 34]. To our knowledge, no studies have attempted to culture *Bartonella* from the blood of healthy humans in Spain. Although a study from the United States found a statistical association between *Bartonella* spp. bacteremia and symptomatology [35], that study, the study presented here, and the Brazilian blood donor study indicate that *B. henselae* bacteremia can be documented in asymptomatic individuals.

Serology can only imply that the investigated population has been exposed to one or more *Bartonella* spp., whereas PCR amplification and DNA sequence confirmation of bacterial DNA from blood or cultures supports infection at the time of sampling. PCR and DNA sequencing confirmed the infection with *B. henselae*, *B. quintana*, and two *B. v*. *berkhoffii* genotypes (I and III, respectively). Four out of seven infected individuals were asymptomatic at the time of sample collection and the remaining three reported historical symptoms such as insomnia, memory problems and fatigue. *B. v*. *berkhoffii* genotype III has been recently documented as a cause of endocarditis and myocarditis in dogs [36], and genotype II was isolated from a boy with epitheliod hemangioendothelioma [37]. *Bartonella v*. *berkhoffii* has been involved in at least one human case of endocarditis [38], and it has also been found in patients with neurological disorders [39] and rheumatic symptoms [40]. A case of perinatal transmission of both *B. henselae* and *B. v*. *berkhoffii* has also been reported [41]. Although canines are the primary reservoir hosts for *B. v*. *berkhoffii,* the vector(s) responsible for transmission of this infection are unknown [42, 43].

Unexpectedly, two asymptomatic veterinarians were enrichment blood culture/PCR-positive to *B. quintana*, the agent of trench fever and other diseases. It is known that, outside of the context of HIV, *B. quintana* has been isolated from afebrile homeless and alcoholic patients from North America and small European clusters [15], but to our knowledge never before from a healthy individual. The mode of *B. quintana* acquisition in these two individuals is unresolved, as none of the study subjects recalled exposure to body lice (*Pediculus humanus corporis*), considered the sole primary vectors of *B. quintana.* These arthropods are common under poor hygiene conditions, which was not the case of the participants of our study. Apart from body lice, *B. quintana* DNA has been amplified from cat fleas and bed bugs (*Cimex lectularius*) [44, 45]. *B. quintana* endocarditis has been documented in dogs from New Zealand and the United States [46], and bites or scratches from a feral cat were implicated as the source of *B. quintana* transmission to a previously healthy, bacteremic human [47]. Collectively, these findings suggest that other arthropods, cats, dogs, or other animals could have been the source of *B. quintana* infections since the individuals reported exposure to fleas, head lice (*Pediculus humanus capitis*) and a variety of animals.

To a substantial degree, the current re-emergence or rediscovery of *Bartonella* spp. evolved as a result of the detection of *B. quintana* and *B. henselae* infections in HIV patients with bacillary angiomatosis or peliosis hepatis. Thus, infection in severely immunocompromised individuals was required before the worldwide clinical emergence of the genus *Bartonella* became recognized. During the ensuing decades, there have been continuous and substantial changes in our understanding of the epidemiology, pathophysiology and vector biology associated with these bacteria.

## Conclusions

This study documented high serological and molecular prevalence of *Bartonella* in veterinary personnel. Future research is required to clarify the duration of and potential relationship to *Bartonella* spp. bacteremia and non-specific symptoms or subtle pathology in immunocompetent individuals.

## Additional files

Additional file 1: Table S1. Exposures and demographics of veterinary subjects, tests statistics and *P*-values for the differences between *Bartonella* PCR-positive and PCR-negative individuals. (DOCX 20 kb)

Additional file 2: Table S2. Exposures, demographics and clinical features, if any, of bacteremic veterinary personnel based upon BAPGM enrichment blood culture/PCR positivity. (DOCX 17 kb)

## Abbreviations

ATCC: American Type Culture Collection
*B. henselae* H1: *Bartonella henselae* Houston 1 strain
*B. henselae* SA2: *Bartonella henselae* San Antonio 2 strain
BAPGM: *Bartonella* alphaproteobacteria growth medium
BCN: Blood culture negative
CSD: Cat scratch disease
DH82: A canine monocytoid cell line
EDTA: Ethylenediaminetetraacetic acid
HIV: Human immunodeficiency virus
IFA: Immunofluorescence antibody assay
IgG: Immunoglobulin class G
ITS: Internal transcribed spacer
PBS: Phosphate-buffered saline
