# Supplementary material for: Prevalence of Bartonella spp. by culture, PCR and serology, in veterinary personnel from Spain
Source: Parasit Vectors. 2017 Nov 7;10:553. doi: 10.1186/s13071-017-2483-z (PMC5678790; doi:10.1186/s13071-017-2483-z)
Supplement: Supplementary file 1 — Exposures and demographics of veterinary subjects, tests statistics and P-values for the differences between Bartonella PCR-positive and PCR-negative individuals. (DOCX 20 kb) [file 13071_2017_2483_MOESM1_ESM.docx]

Supplementary Table 1.- Exposures and demographics of veterinary subjects, tests statistic and p-values of differences between *Bartonella* PCR positive and PCR negative individuals.

| **Demographics and travel** | **n=89 (%)** | **U / OR** | **CI 95%** | **p-values** |
| --- | --- | --- | --- | --- |
| Age (years)  Median  Mean  Minimum  Maximum | 44.5  42.0  22.0  61.0 | U = 251.5 |  | 0.627 |
| Years in practice  Median  Mean  Minimum  Maximum | 20.0  16.6  0.2  35.0 | U = 43.5 |  | 0.185 |
| **Gender**  Female  Male | 49 (55.1%)  40 (44.9%) | 0.187 | 0.004 - 1.644 | 0.124 |
| **Housing**  Urban  Peri-urban  Rural area/Farm  Rural area/Forest | 54 (60.7%)  24 (27.0%)  7 (7.9%)  2 (2.2%) |  |  | 0.635 |
| **Clinical condition***  Healthy  Persistent / Chronic disease  Infectious disease | 76 (85.4%)  26 (29.2%)  6 (6.7%) | 0.393  1.908  7.453 | 0.055 - 4.603  0.259 – 12.258  0.551 – 69.885 | 0.270  0.412  0.069 |
| **Clinical features**  Persistent fever  Fatigue  Insomnia  Sleepiness  Memory problems  Headache  Irritability  Anxiety  Depression  Tremor  Vision impairment  Eye pain  Balance problems  Bladder dysfunction  Shortness of breath  Tachycardia  Poor appetite  Weight gain  Chronic diarrhea  Corticosteroid treatment | 1 (1%)  16 (18.0%)  18 (20.2%)  8 (9.0%)  11 (12.4%)  23 (25.8%)  10 (11.2%)  4 (4.5%)  2 (2.2%)  1 (1.1%)  3 (3.4%)  4 (4.5%)  2 (2.2%)  3 (3.4%)  4 (4.9%)  7 (7.9%)  2 (2.2%)  10 (11.2%)  6 (6.7%)  7 (7.9%) | ∞  3.897  3.291  1.772  6.684  2.300  1.347  0.000  0.000  ∞  0.000  0.000  0.000  6.389  0.000  2.088  0.000  3.620  0.000  0.000 | 0.300 - ∞  0.511 – 26.153  0.437 – 21.783  0.035 – 18.444  0.833 – 48.096  0.310 – 14.890  0.026 – 13.326  0.000 – 19.922  0.000 – 66.007  0.300 - ∞  0.000 – 31.193  0.000 – 19.922  0.000 – 66.007  0.097 – 140.432  0.000 – 19.922  0.039 – 22.632  0.000 – 66.007  0.299 – 27.217  0.000 – 11.337  0.000 – 9.258 | 0.079  0.107  0.144  0.493  0.051  0.369  0.579  1  1  0.079  1  1  1  0.220  1  0.448  1  0.176  1  1 |
| **Allergy**  Autoimmune disease  Dried fruits  Metals  Food  Animals  Lactose  Mites  Pollen | 18 (20.2%)  0 (0%)  0 (0%)  1 (1.1%)  3 (3.4%)  7 (8.5%)  1 (1.1%)  2 (2.2%)  4 (4.5%) | 1.640  -  -  0.000  0.000  0.000  0.000  0.000  0.000 | 0.144 – 11.200  -  -  0.000 – 453.626  0.000 – 31.193  0.000 – 9.258  0.000 – 453.626  0.000 – 66.007  0.000 – 19.922 | 0.626  -  -  1  1  1  1  1  1 |
| **Pets**  Dogs  Cats  Birds | 84 (94.4%)  76 (85.4%)  62 (69.7%)  32 (36.0%) | ∞  1.028  1.095  0.277 | 0.069 - ∞  0.108 – 51.205  0.165 – 12.240  0.006 – 2.455 | 1  1  1  0.414 |
| **Arthropod exposure**  Fleas  Ticks  Lice  Bed bugs | 74 (83.1%)  66 (74.2%)  29 (32.6%)  4 (4.5%) | 1.233  0.862  1.606  0.000 | 0.132 – 60.811  0.129 – 9.699  0.219 – 10.257  0.000 – 19.922 | 1  1  0.678  1 |
| **Animal exposure**  Dogs  Cats | 72 (79.1%)  70 (78.7%) | ∞  ∞ | 0.339 - ∞  0.393 - ∞ | 0.338  0.338 |
| **Animal scratches and/or bites**  Dogs  Cats  Birds | 76 (85.4%)  75 (84.3%)  21 (23.6%) | ∞  ∞  0.000 | 0.239 - ∞  0.263 - ∞  0.000 – 2.216 | 0.588  0.591  0.192 |
| **Outdoors activities**  Trekking  Hunting  Fishing  Agriculture  Gardening | 46 (51.7%)  3 (3.4%)  1 (1.1%)  7 (7.9%)  26 (29.2%) | 2.476  0.000  ∞  2.088  0.967 | 0.379 – 27.411  0.000 – 31.193  0.300 - ∞  0.039 – 22.632  0.086 – 6.423 | 0.436  1  0.079  0.448  1 |
| **Travel out of Spain**  Other European countries  North America  Central America  South America  Asia  Africa  Australia/New Zealand | 63 (70.8%)  21 (23.6%)  21 (23.6%)  14 (15.7%)  12 (13.5%)  18 (20.2%)  3 (3.4%) | 1.034  0.520  0.520  0.000  0.000  0.000  0.000 | 0.156 – 11.572  0.011 – 4.700  0.011 – 4.700  0.000 – 3.809  0.000 – 4.645  0.000 – 2.736  0.000 – 31.193 | 1  1  1  0.591  0.587  0.337  1 |

- Percentages may not total 100% if participants checked more than one category.
