# Supplementary material for: Prevalence of Bartonella spp. by culture, PCR and serology, in veterinary personnel from Spain
Source: Parasit Vectors. 2017 Nov 7;10:553. doi: 10.1186/s13071-017-2483-z (PMC5678790; doi:10.1186/s13071-017-2483-z)
Supplement: Supplementary file 2 — Exposures, demographics and clinical features, if any, of bacteremic veterinary personnel based upon BAPGM enrichment blood culture/PCR positivity. (DOCX 17 kb) [file 13071_2017_2483_MOESM2_ESM.docx]

Supplementary Table 2: Exposures, demographics and clinical features, if any, of bacteremic veterinary personnel based upon BAPGM enrichment blood culture/PCR positivity.

| ***Bartonella* sp. by BAPGM+PCR** | **Age / Years in practice** | **Gender** | **Health status** | **Reactive by serologic assays (IFA)** | **Living area** | **Cat exposure** | **Dog exposure** | **Other animal exposure** | **Outdoors activities** | **Arthropod exposure** |
| --- | --- | --- | --- | --- | --- | --- | --- | --- | --- | --- |
| ***Bq*** | 50 / 6 | F | Healthy | *Bvb, Bq, Bk* | Urban | Yes (bite) | Yes (bite) | Bird-Rabbit | Trekking  Gardening | Head lice-Fleas-Ticks-Biting flies-mosquitoes-spiders |
| ***Bvb* TIII** | 36 / N.A. | F | Insomnia-memory problems-fatigue- | *Bvb, Bh, Bk* | Periurban | Yes (bite) | Yes (bite) | Horse | Trekking | Fleas  Ticks |
| ***Bvb* TIII** | 34 / N.A. | F | Insomnia-memory problems-fatigue- | *Bvb, Bk* | Rural (farm) | Yes (bite) | Yes (bite) | Reptile  Rabbit  Rodent | Trekking  Gardening  Agriculture | Head lice-Fleas-Ticks-Biting flies-mosquitoes-spiders |
| ***Bh*** | 37 / 14 | F | Healthy | *Bvb, Bk* | Urban | Yes (bite) | Yes (bite) | Rabbit  Rodent |  | Head lice-Fleas-Ticks-Biting flies-mosquitoes-spiders |
| ***Bq*** | 54 / N.A. | F | Insomnia-memory problems-fatigue-headache-bladder dysfunction-arthralgia | *Bvb, Bq, Bh, Bk* | Periurban | Yes (bite) | Yes(bite) | - | Trekking | Fleas |
| ***Bh*** | 38 / 10 | F | Healthy | Non-reactive | Urban | Yes (bite) | Yes (bite) | Squirrel  Rodent | - | Fleas  Ticks |
| ***Bvb* TI** | 34 / N.A. | M | Healthy | *Bvb, Bh, Bq, Bk* | Urban | Yes (bite) | Yes (bite) | Rabbit | Trekking  Fishing | N.A. |

F: female; M: male; *Bq*: *Bartonella quintana*; *Bvb* TIII: *Bartonella vinsonii* subsp. *berkhoffii* genotype III; *Bh*: *Bartonella henselae; Bvb* TI: *Bartonella vinsonii* subsp. *berkhoffii* genotype I; N.A.: Data not available.
